# United States Veterinary Medical Association

**Published:** 1891-01

**Authors:** Rush S. Huidekoper, W. Horace Hoskins

**Affiliations:** President; Secretary, 12 S. 37th St., Philada.


### UNITED STATES VETERINARY MEDICAL ASSOCIATION.

As the duties of General Secretary have become so multiplied and onerous, it is desirous that in the future all committees and members of the profession desiring information or other matter pertaining to the Association will apply for the same through the Assistant State Secretaries. All those desiring to become members of the Association are recommended to apply through their respective State Secretaries, as their approval of their credentials will greatly facilitate the work of the Comita Minora. Blank applications for membership will hereafter be furnished, and must be properly filled and filed before an applicant's name can be considered. All future certificates of the Association will only be granted after the member-elect has filled and filed certificate of his willingness to sign and uphold the Constitution and

By-laws, a copy of which will be mailed with each certificate, that the members may become fully acquainted with the requirements of the Association.

It is earnestly desired that all members-elect who have not qualified, will do so on or before January 15th, 1891, as the new list of officers, committees and members will be placed in the hands of the printer at that date. Notice is given that no names will be placed in the list of qualified members save those who have complied with the requirements of membership.

RUSH S. HUIDEKOPER, *President.*

W. HORACE HOSKINS, *Secretary,* 12 S. 37th St., Philada.

